# *In vitro* and *in vivo* preclinical venom inhibition assays identify metalloproteinase inhibiting drugs as potential future treatments for snakebite envenoming by *Dispholidus typus*

**DOI:** 10.1016/j.toxcx.2022.100118

**Published:** 2022-06

**Authors:** Stefanie K. Menzies, Rachel H. Clare, Chunfang Xie, Adam Westhorpe, Steven R. Hall, Rebecca J. Edge, Jaffer Alsolaiss, Edouard Crittenden, Amy E. Marriott, Robert A. Harrison, Jeroen Kool, Nicholas R. Casewell

**Affiliations:** a Centre for Snakebite Research and Interventions, Department of Tropical Disease Biology, Liverpool School of Tropical Medicine, Pembroke Place, Liverpool, L3 5QA, England, UK; b Centre for Drugs and Diagnostics, Department of Tropical Disease Biology, Liverpool School of Tropical Medicine, Pembroke Place, Liverpool, L3 5QA, England, UK; c Amsterdam Institute of Molecular and Life Sciences, Division of BioAnalytical Chemistry, Department of Chemistry and Pharmaceutical Sciences, Faculty of Science, Vrije Universiteit Amsterdam, De Boelelaan 1085, 1081HV, Amsterdam, the Netherlands

**Keywords:** Small molecules, Drugs, Boomslang, Snakebite, SVMP, SVMP, snake venom metalloproteinase, VICC, venom-induced consumption coagulopathy

## Abstract

Snakebite envenoming affects more than 250,000 people annually in sub-Saharan Africa. Envenoming by *Dispholidus typus* (boomslang) results in venom-induced consumption coagulopathy (VICC), whereby highly abundant prothrombin-activating snake venom metalloproteinases (SVMPs) consume clotting factors and deplete fibrinogen. The only available treatment for *D. typus* envenoming is the monovalent SAIMR Boomslang antivenom. Treatment options are urgently required because this antivenom is often difficult to source and, at US$6000/vial, typically unaffordable for most snakebite patients. We therefore investigated the *in vitro* and *in vivo* preclinical efficacy of four SVMP inhibitors to neutralise the effects of *D. typus* venom; the matrix metalloproteinase inhibitors marimastat and prinomastat, and the metal chelators dimercaprol and DMPS*.* The venom of *D. typus* exhibited an SVMP-driven procoagulant phenotype *in vitro*. Marimastat and prinomastat demonstrated equipotent inhibition of the SVMP-mediated procoagulant activity of the venom *in vitro*, whereas dimercaprol and DMPS showed considerably lower potency. However, when tested in preclinical murine models of envenoming using mixed sex CD1 mice, DMPS and marimastat demonstrated partial protection against venom lethality, demonstrated by prolonged survival times of experimental animals, whereas dimercaprol and prinomastat failed to confer any protection at the doses tested. The preclinical results presented here demonstrate that DMPS and marimastat show potential as novel small molecule-based therapeutics for *D. typus* snakebite envenoming. These two drugs have been previously shown to be effective against *Echis ocellatus* VICC in preclinical models, and thus we conclude that marimastat and DMPS should be further explored as potentially valuable early intervention therapeutics to broadly treat VICC following snakebite envenoming in sub-Saharan Africa.

## Highlights

• Snake venom metalloproteinases mediate pathology of *Dispholidus typus* envenoming.
• Small molecule drugs were tested for inhibition of *D. typus* venom *in vitro* and *in vivo*.
• Performance in *in vitro* experiments did not predict *in vivo* efficacy.
• Marimastat and DMPS show potential as novel therapeutics for *D. typus* envenomation.

## Graphical abstract

**Figure:**
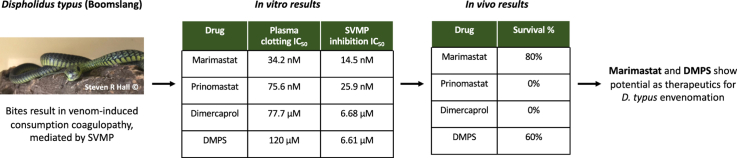

## 1 Introduction

More than 250,000 cases of snakebite envenoming are estimated to occur annually in sub-Saharan Africa (sSA) (Halilu et al., 2019), disproportionately affecting those in rural, impoverished communities without adequate access to healthcare (Longbottom et al., 2018; Harrison et al., 2019). Venom-induced consumption coagulopathy (VICC) is a common manifestation of snakebite envenoming, during which procoagulant venom toxins consume clotting factors resulting in the ensuing depletion of fibrinogen and, ultimately, coagulopathy (Berling and Isbister, 2015). Several clotting factors are the target for procoagulant snake venom toxins, and these include Factor X, Factor V, fibrinogen and prothrombin (Berling and Isbister, 2015). While infrequent, envenomings by the rear fanged African colubrid *Dispholidus typus* (boomslang) are characterised by causing VICC (Lakier and Fritz, 1969; Matell et al., 1973; Gomperts and Demetriou, 1977). The venom of this species is known to potently activate prothrombin (Debono et al., 2017), resulting in the liberation of thrombin, and the subsequent downstream consumption of fibrinogen and fibrin, causing dysregulation of coagulation (Debono et al., 2017, 2020; Ainsworth et al., 2018). The activation of prothrombin is likely the result of snake venom metalloproteinases (SVMPs) (Ainsworth et al., 2018; Debono et al., 2020), which are the dominant toxin type present in the venom and account for almost 75% of the proteinaceous toxins (Pla et al., 2017; Kamiguti et al., 2000). Other minor toxin families identified in the *D. typus* venom proteome (each constituting <10% of the venom proteome) include three-finger toxins, phospholipases A_2_ (PLA_2_s), cysteine-rich secretory proteins (CRISPs), snake venom serine proteases (SVSPs) and C-type lectin-like toxins (Pla et al., 2017), though their contribution to envenoming pathology remains unclear.

*Dispholidus typus* is broadly distributed throughout much of sub-Saharan Africa and whilst incidences of envenoming are rare, the rapid and severe VICC consequences pose considerable clinical challenges. This is because the only specific treatment for *D. typus* envenoming is the monospecific F(ab’)_2_ antivenom “SAIMR Boomslang” (South African Vaccine Producers Pty Ltd), which has limited availability outside of the Southern Africa Economic Community, and costs as much as US$6050 per vial (Krüger and Lemke, 2019). Given that *D. typus* exhibits a broad geographical distribution throughout much of sub-Saharan Africa, the only specific treatment for envenomings caused by this species is largely unobtainable for snakebite victims who either cannot afford, or do not have access to the antivenom (Ainsworth et al., 2018), and thus investigating novel treatments is a research priority.

More generally, it is well recognised that conventional polyclonal antibody-based antivenoms have several shortcomings, despite being life-saving therapeutics. In addition to often being unaffordable to many snakebite victims, they are associated with high rates of adverse reactions (de Silva et al., 2016; Potet et al., 2019), and have poor dose efficacy, with only ∼10–20% of the active immunoglobulins recognising and binding to venom toxins (Casewell et al., 2010). Logistically, antivenoms are poorly suited for the rural locations in which they are typically required; for example many antivenoms rely on cold chain transport and storage and must be administered intravenously by trained staff in healthcare facilities (World Health Organization, 2010). Indeed, up to 75% of deaths from snakebite are estimated to occur before patients are able to reach healthcare facilities, thus there is a compelling need to identify novel snakebite treatments that could be administered in the community soon after a bite (Bulfone et al., 2018).

To this end, small molecule-based drugs (i.e. ‘toxin inhibitors’) have received considerable interest as novel snakebite therapeutics, both as individual treatments or in combination with existing antivenoms (Bulfone et al., 2018; Williams et al., 2019; Clare et al., 2021). Small molecule drugs have a number of potentially advantageous characteristics over antivenoms, including improved affordability and stability, oral delivery format, higher tolerability (Clare et al., 2021), and improved tissue penetration (Rucavado et al., 2000; Layfield et al., 2020). Previously, Ainsworth et al. demonstrated the *in vitro* inhibitory effect of the metal chelator EDTA against SVMP-mediated prothrombin activation caused by *D. typus* venom, suggesting that small molecule inhibitors may be effective therapeutics for *D. typus* envenoming (Ainsworth et al., 2018). In the same study, Ainsworth et al. demonstrated in a murine preclinical model that EDTA was protective against the lethal effects of *Echis ocellatus* venom, an African viper which, similar to *D. typus* venom, contains a high abundance of SVMP toxins (Ainsworth et al., 2018), including prothrombin activators, and causes VICC in envenomed victims (Warrell et al., 1977; Arias et al., 2017). Other small molecule drugs with SVMP-inhibiting potential include other metal chelators, such as dimercaprol and DMPS (2,3-dimercapto-1-propanesulfonic acid) (Xie et al., 2020a, 2020b; Albulescu et al., 2020a), and the mimetic matrix metalloproteinase inhibitors marimastat, batimastat and prinomastat (Layfield et al., 2020; Arias et al., 2017; Howes et al., 2007). Marimastat and batimastat were found to effectively inhibit SVMP activity and reduce haemorrhagic pathologies in murine models of *E. ocellatus* envenoming (Arias et al., 2017), and inhibit the procoagulant effects of several viper venoms *in vitro*^24,25^*.* Similarly, prinomastat (AG-3340) showed inhibitory activity against the haemorrhagic effects of both purified SVMPs and the crude venom of *E. ocellatus*^28^*.* The metal chelators dimercaprol and DMPS have also been shown to inhibit SVMP activity of *E. ocellatus* venom *in vitro,* with DMPS also demonstrating *in vivo* preclinical neutralisation of venom lethality and haemorrhage (Albulescu et al., 2020a). While *D. typus* venom is abundant with SVMPs, PLA_2_ toxins are also thought to contribute to the coagulopathy induced by this venom (Slagboom et al., 2020). The small molecule drug varespladib has been extensively investigated as an inhibitor of venom PLA_2_ toxins found in a range of snake species, with such studies showing potent neutralisation of PLA_2_ activity (Xie et al., 2020a, 2020b, 2020c) and associated anticoagulant, haemorrhagic, myotoxic and neurotoxic pathologies (Wang et al., 2018; Bryan-Quirós et al., 2019; Bittenbinder et al., 2018; Lewin et al., 2018a, 2018b). Thus, small molecule treatments for snakebite have the potential to overcome the species-specific and geographically-restrictive limitations associated with current antivenom.

Despite these promising recent research outcomes, further investigation is required to explore the inhibitory breadth and potency of small molecule toxin inhibitors due to the ubiquitous variability in snake venom composition and therefore, also, the variant toxin specificities of these inhibitory small molecule drugs. In particular, despite overarching similarities in venom composition and ensuing snakebite pathology between *Echis* spp. and *D. typus*^9^*,* the SVMPs of *D. typus* have evolved their prothrombin activating ability independently of those found in the venom of *Echis* spp. (Debono et al., 2020). Consequently, building on previous principles demonstrated for *Echis* spp. (Arias et al., 2017; Xie et al., 2020a; Albulescu et al., 2020a, 2020b), in this study four small molecule drugs were investigated *in vitro* and *in vivo* to assess their inhibitory potential against the venom of *D. typus*. To do so, we applied *in vitro* metalloproteinase and coagulation bioassays on crude and nanofractionated venom, and *in vivo* murine models of envenoming to assess neutralisation of venom lethality.

## 2 Methods

### 2.1 Venoms

Lyophilised *D. typus* venom (Product code L1403, origin South Africa, purity >99%) was sourced from Latoxan (Portes les Valence, France) and stored at 4 °C to ensure long-term stability. Prior to use, venom was resuspended in PBS (pH 7.4, Gibco) at 1 mg/mL for *in vitro* experiments and 5 mg/mL for *in vivo* experiments.

### 2.2 Drug preparations for *in vitro* studies against crude venom

The small molecule SVMP inhibitors tested were; dimercaprol (2,3-dimercapto-1-propanol, ≥98% iodometric, Cat no: 64,046, Sigma), DMPS (2,3-dimercapto-1-propane-sulfonic acid sodium salt monohydrate, 98%, Cat no: H56578, Alfa Aesar), marimastat ((2*S*,3*R*)-*N*4-[(1*S*)-2,2-Dimethyl-1-[(methylamino)carbonyl]propyl]-*N*1,2-dihydroxy-3-(2-methylpropyl)butanediamide, >98%, Cat no: 2631, Tocris Bioscience), prinomastat hydrochloride (Cat no: HY-12170A, >98%, MedChemExpress). Varespladib (2-[[3-(2-Amino-2-oxoacetyl)-2-ethyl-1-(phenylmethyl)-1H-indol-4-yl]oxy]-acetic acid, Cat no: SML1100, >98% HPLC, Sigma) was used as a small molecule drug control. All drugs were reconstituted in dimethyl sulfoxide (DMSO) (Sigma) to 10 mM stocks and stored at −20 °C. Daughter plates were created at 1 mM concentrations in 384-well format to allow the creation of assay-ready plates using a VIAFLO 384 electronic pipette (Integra). Daughter plates and assay-ready plates were stored at −20 °C, with the latter used within a month of creation. For the SVMP assay 0.91 μL of each drug was plated (final reaction volume of 91 μL), while 0.5 μL was plated for the coagulation assay (final reaction volume of 50 μL). For marimastat, prinomastat and varespladib, dose response curves were created at a final concentration range of 10 μM to 4.8 pM using a two-fold dilution (50 μL drug into 50 μL of DMSO), with each concentration tested in duplicate. For DMPS and dimercarpol, dose response curves were created at a final concentration range of 160 μM to 76.3 pM using a two-fold dilution (50 μL drug into 50 μL of DMSO), with each concentration tested in duplicate.

### 2.3 *In vitro* neutralisation of coagulopathic crude venom activity

To assess the inhibitory potency of the selected drugs against coagulopathic venom activity we used a previously described absorbance-based plasma clotting assay (Still et al., 2017). Citrated bovine plasma (VWR) was defrosted and centrifuged at 858×*g* for 5 minutes to remove precipitates before use. Thereafter, 100 ng of venom in 10 μL PBS was added to each well in the 384-well assay-ready plate (containing 0.5 μL of 1 mM of inhibitor) using a VIAFLO 384, the plate was then briefly spun down in a Platefuge (Benchmark Scientific) and incubated at 37 °C for 25 min, followed by a further 5 min acclimatisation at room temperate. Next, 20 μL of 20 mM CaCl_2_ was added using a MultiDrop 384 Reagent Dispenser (ThermoFisher Scientific), followed by the immediate addition of 20 μL citrated bovine plasma. The plate was then immediately read for kinetic absorbance at 595 nm for 116 min using a FLUOstar Omega platereader (BMG Labtech).

Assays were performed in triplicate and each assay contained technical duplicates at each dose. Positive control values were generated using DMSO + venom, and negative control values were generated using DMSO in the absence of venom. All compounds were analysed for their ability to return clotting to normal at the timepoint at which the positive and negative absorbance values were furthest apart. For this, the raw values were normalised to show percentage of normal clotting, e.g. a value of 100% meant the compound returned clotting to that of the negative control. These percentage values were plotted and fitted with a nonlinear regression curve for the normalised response (variable slope) to calculate the IC_50_ data and 95% confidence intervals for each compound using GraphPad Prism 9.0 (GraphPad Software, San Diego, USA). Multiple comparisons one-way ANOVA test was used to compare IC_50_ values generated for each replicate plate, using GraphPad Prism 9.0.

### 2.4 Venom nanofractionation

To further explore the inhibitory specificity of the selected drugs, we fractionated *D. typus* into toxin constituents and repeated the plasma bioassay. Venom nanofractionation (Slagboom et al., 2020; Zietek et al., 2018) was performed on a UPLC system ('s Hertogenbosch, The Netherlands) controlled by Shimadzu Lab Solutions software. Venom solution was prepared by dissolving lyophilised *D. typus* venom into water (purified by Milli-Q Plus system, Millipore) to a concentration of 5.0 mg/mL and stored at −80 °C until use. For each analysis, 50 μL venom solution (1.0 mg/mL) was injected by a Shimadzu SIL-30AC autosampler after diluting the stock venom solutions (5.0 ± 0.1 mg/mL) in Milli-Q water. A Waters XBridge reversed-phase C18 column (4.6 × 100 mm column with a 5 μm particle size and a 300 Å pore size) was used for gradient separation at 30 °C. Mobile phase A was composed of 98% water, 2% acetonitrile (ACN) (Biosolve) and 0.1% formic acid (FA) (Biosolve), while mobile phase B was composed of 98% ACN, 2% water and 0.1% FA. The total solvent flow rate was maintained at 0.5 mL/min and the gradients were run as follows: linear increase of eluent B from 0 to 50% in 20 min followed by a linear increase to 90% B in 4 min, then isocratic elution at 90% for 5 min, subsequently the eluent B was decreased from 90% to 0% in 1 min followed by an equilibration of 10 min at 0% B. The column effluent was split as two parts (9:1), with the smaller fraction (10%) sent to a Shimadzu SPD-M20A prominence diode array detector. The larger fraction (90%) was directed to a FractioMate nanofractionator (SPARK-Holland & VU) and fractions collected onto transparent 384-well plates (F-bottom, rounded square well, polystyrene, without lid, clear, non-sterile; Greiner Bio One). The nanofractionator was controlled by FractioMator software (Spark-Holland) to collect fractions continuously at a resolution of 6 s/well. After collection, the well plates with venom fractions were dried overnight in a Christ Rotational Vacuum Concentrator (RVC 2–33 CD plus, Zalm en Kipp, Breukelen, The Netherlands), to remove any solvent remaining in the wells. The vacuum concentrator was equipped with a cooling trap maintained at −80 °C during operation. The dried plates were then stored at −20 °C until bioassaying. Note that during reversed-phase separation, some toxins might denature during separation conditions (i.e. due to FA and/or ACN). This unfortunately cannot be circumvented as the FA is required to achieve sufficient separation resolution and ionisation efficiency in mass spectrometry. This study limitation has been discussed thoroughly elsewhere (Slagboom et al., 2020).

### 2.5 *In vitro* neutralisation of coagulopathic venom toxin fractions

The small molecule inhibitors marimastat ((2S,3R)-N4-[(1S)-2,2-Dimethyl-1-[(methylamino)carbonyl] propyl]-N1,2-dihydroxy-3-(2-methylpropyl) butanediamide), prinomastat hydrochloride (AG-3340 hydrochloride), dimercaprol (2,3-Dimercapto-1-propanol), DMPS (2,3-dimercapto-1-propane-sulfonic acid sodium salt monohydrate) and varespladib (A-001) were purchased from Sigma-Aldrich. Bovine plasma (Sodium Citrated, Sterile Filtered, Product Code: S0260) was purchased from Biowest. For assay preparation, the CaCl_2_ (Biosolve), which was used to de-citrate plasma to initiate coagulation in the coagulation assay, was dissolved in Milli-Q water to 20 mM. The inhibitors were dissolved in DMSO (≥99.9%, Sigma-Aldrich) to a concentration of 10 mM and stored at −20 °C. The plasma was defrosted and then centrifuged at 805×*g* for 4 min in a 5810 R centrifuge (Eppendorf) to remove possible particulate matter. The inhibitor stock solutions were diluted in PBS buffer to the described concentrations, then 10 μL of each diluted inhibitor solution was pipetted to all wells of plate containing freeze-dried nanofractionated venom fractions by a VWR Multichannel Electronic Pipet, followed by centrifuging the plate for 1 min at 805×*g*. Next, a pre-incubation step for 30 min at room temperature was performed. Final concentrations of inhibitor solutions used for the coagulation bioassay were 20, 4, 0.8, 0.16 and/or 0.032 μM (with corresponding DMSO final concentrations of 0.02%, 0.004%, 0.0008%, 0.00016% and 0.000032%, respectively). After this incubation step, the HTS coagulation assay was performed as described by Still et al. (2017). A Multidrop 384 Reagent Dispenser (Thermo Fisher Scientific) was used to dispense 20 μL of CaCl_2_ solution onto all wells of the 384-well plates, followed by 20 μL plasma after rinsing of the Multidrop with deionized water between dispensing. Kinetic absorbance measurements were conducted immediately for 100 min at 595 nm at 25 °C using a Varioskan Flash Multimode Reader (Thermo Fisher Scientific). Venom-only analyses were performed as control experiments, for which 10 μL PBS instead of inhibitor solution was added to all wells of the vacuum centrifuge-dried nanofractionated plates. Each nanofractionation analysis was performed in at least duplicate.

The resulting coagulation chromatograms were plotted as described by Slagboom et al. (2020), with each chromatogram reconstructed to display ‘very fast coagulation’, ‘slightly/medium increased coagulation’ and ‘anticoagulation’. To plot the very fast coagulation chromatogram, the average slope of the first 5 min in the assay was plotted, and for the slightly/medium coagulation chromatogram the average slope of the first 20 min was plotted. For anticoagulant chromatogram the final (end-point) read at 100 min was plotted. Clotting velocities were all plotted against the venom nanofractionation time, producing positive peaks for procoagulant compounds and negative peaks for anticoagulant compounds.

### 2.6 *In vitro* neutralisation of venom SVMP activity

The SVMP activity of crude *D. typus* venom in the presence of inhibitors or vehicle control (DMSO), was measured using a quenched fluorogenic substrate (ES010 Mca-KPLGL-Dpa-AR-NH2, R&D Biosystems; substrate for matrix metalloprotease (MMP) −1, −2, −7, −8, −9, −12, −13, −14, −15, −16, a disintegrin and metalloproteinase (ADAM)10, ADAM17/TACE, Cathepsin D and Cathepsin E), in line with principles previously described (Albulescu et al., 2020a). The substrate was suspended in reaction buffer (150 mM NaCl, 50 mM Tris-HCl pH 7.5) and used at a final concentration of 10 μM (supplied as a 6.2 mM stock). Reactions consisted of 1 μg of venom (1 μg in 15 μL PBS) co-incubated with 0.91 μL of 1 mM of inhibitor. The 384 well plate (Greiner) was briefly spun down in a Platefuge (Benchmark Scientific) and incubated at 37 °C for 25 min, with an additional 5 min acclimatisation at room temperate, before the final addition of the freshly diluted fluorogenic substrate (75 μL of 12.1 μM). The plate was immediately run on a CLARIOstar platereader (BMG Labtech) at an excitation wavelength of 320–10 nm and emission wavelength of 420–10 nm with 10 flashes per well at 25 °C for 100 cycles (each cycle time 79 s). The assay was performed independently in duplicate. The end-reads were calculated for each sample at the time where all fluorescence curves had typically reached a plateau (maximum fluorescence). SVMP activity was calculated for each test condition as a percentage of the mean of the DMSO only wells (100% activity), with a baseline of the marimastat 10 μM controls representing 0% activity. IC_50_ values were calculated from the percentage inhibition values by fitting a nonlinear regression curve for the normalised response (variable slope) for each compound using GraphPad Prism 9.0 (GraphPad Software, San Diego, USA). The best-fit IC_50_ values for each replicate were compared to identify significant differences between the IC_50_ values for each drug using one-way multiple comparisons ANOVA analysis in GraphPad Prism 9.0.

### 2.7 *In vivo* neutralisation of venom lethality

#### 2.7.1 Animal ethics

All animal experiments were performed using protocols approved by the Animal Welfare and Ethical Review Boards of the Liverpool School of Tropical Medicine and the University of Liverpool, under project licence (P58464F90) approved by the UK Home Office in accordance with the UK Animal (Scientific Procedures) Act 1986.

#### 2.7.2 Animal maintenance

CD1 mice (18–20 g) were sourced from Charles River (UK) and acclimatised for a minimum of one week before experimentation. Male mice were used for all experiments except for the prinomastat drug only control group. Mice were grouped in cages of five, with room conditions of approximately 22 °C at 40–50% humidity, with 12/12 hour light cycles, and given *ad lib* access to CRM irradiated food (Special Diet Services, UK) and reverse osmosis water in an automatic water system. Mice were housed in specific-pathogen free facilities in Techniplast GM500 cages containing Lignocell bedding (JRS, Germany), Sizzlenest zigzag fibres as nesting material (RAJA), and supplied with environmental enrichment materials.

#### 2.7.3 Co-incubation model of preclinical efficacy

The median murine lethal dose (LD_50_) for *D. typus* venom administered by intravenous injection was previously determined as 22.29 μg per mouse (Ainsworth et al., 2018). To determine the efficacy of small molecule inhibitors against *D. typus* venom, a refined version of the WHO recommended antivenom ED_50_ neutralisation experiments was used, in which ∼4 x LD_50_ doses of venom (90 μg) were pre-incubated with each small molecule inhibitor. Drug stocks were freshly prepared to allow for a ratio of 1:1.33 venom to inhibitor as previously defined by marimastat *in vivo* testing against other snake venoms (Albulescu et al., 2020b). Drugs tested *in vivo* were dimercaprol (2,3-dimercapto-1-propanol ≥98% iodometric, Cat no: 64,046, Sigma-Aldrich), marimastat (>98% HPLC, Cat no: M2699, Sigma-Aldrich), and prinomastat hydrochloride (≥95% HPLC, Cat no: PZ0198, Merck), all resuspended at 1 mg/mL in water, and DMPS (2,3-dimercapto-1-propanesulfonic acid sodium salt monohydrate, 95%, Cat no: H56578, Alfa Aesar) resuspended at 1 mg/mL in PBS. Groups of five mice received experimental doses that consisted of either: (a) venom only (4 x LD_50_ dose) or (b) venom (4 x LD_50_ dose) with drug (118 μg) or (c) drug only (118 μg) to assess drug safety. The control group was the venom only group, against which all drug treatments were compared. Each experimental group comprised five animals as this was previously determined to be the minimum number of animals required to produce statistically significant results (World Health Organization, 2018), although the prinomastat only (no venom) control group contained only four due to the misdosing of one animal. No randomisation was used to allocate experimental groups – mice were randomly allocated into cages of five prior to the experiment, and each cage formed one treatment group. No criteria for including or excluding animals was applied, and all data points were included in analyses. A total of 45 mice were used. All experimental doses were prepared to a volume of 200 μL in PBS and incubated at 37 °C for 30 minutes prior to intravenous injection via the tail vein. Animals were monitored for humane endpoints (loss of righting reflex, seizure, external haemorrhage) for 6 hours, and any animals showing such signs were immediately euthanised by rising concentrations of carbon dioxide. All observations were performed by mixed gender experimenters who were blinded to the drug group allocation. Time of death, number of deaths and number of survivors were recorded, where deaths and times of death represent implementation of humane endpoint-dictated euthanasia. Kaplan-Meier survival plots were generated using GraphPad Prism 9.0 (GraphPad Software, San Diego, USA) and log-rank (Mantel-Cox) tests were used to statistically compare the survival times between groups treated with and without drug.

## 3 Results

### 3.1 Small molecule drugs have varying effects on the procoagulant activity of crude *D. typus* venom

**Fig. 1:**
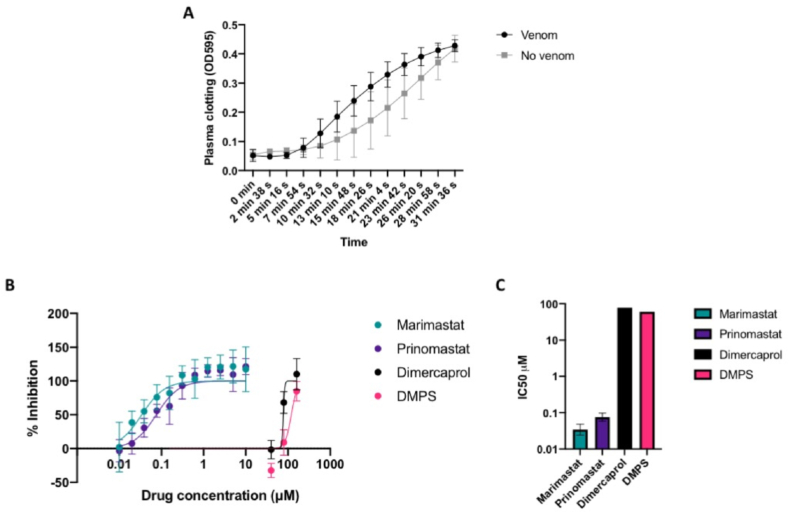
**Effects of *D. typus* venom, and inhibition by small molecule drugs, on *in vitro* plasma clotting measured by absorbance at 595 nm (OD**_**595**_**).A)** Clotting as indicated by the increase in OD_595_ in the presence of crude *D. typus* venom (black circles) compared to normal clotting in the absence of venom (grey squares). Data points represent the mean of twelve individual values recorded over three independent technical replicates, and error bars represent standard deviation. **B)** Inhibitory activity of marimastat (teal circles), prinomastat (dark purple circles), dimercaprol (black circles) and DMPS (pink circles) over a two-fold serial dilution curve, from which IC_50_ values were calculated. Inhibitory activity is expressed as a percentage of normal clotting, where 100% inhibition represents return of clotting to normal plasma clotting levels. Data points represent the mean of six individual values recorded over three independent technical replicates, and error bars represent standard deviation. **C)** Calculated IC_50_ values for marimastat, prinomastat, dimercaprol and DMPS. Data points represent the best fit IC_50_ value and error bars represent 95% confidence intervals (not calculated for dimercaprol and DMPS).

The addition of *D. typus* venom to bovine plasma in the coagulation assay resulted in earlier stimulation of clotting compared to the no venom control (natural clotting), highlighting the procoagulant nature of this venom (Fig. 1A). The effects of the small molecules against the procoagulant activity of crude *D. typus* venom are shown in Fig. 1. Weak venom-inhibitory effects were observed for the metal chelators dimercaprol and DMPS at micromolar concentrations. As shown in Fig. 1B, concentrations of 160 μM showed strong inhibitory activity for both drugs, but this inhibitory effect rapidly decreased at lower concentrations, with no effect observed at concentrations of 10 μM and lower. IC_50_ values were determined to be 77.7 μM for dimercaprol and 120 μM for DMPS (Fig. 1C), although due to the small number of data points between 0 and 100% inhibition these values must be interpreted with caution and 95% confidence intervals were unable to be calculated. Contrastingly, the peptidomimetic matrix metalloproteinase inhibitors marimastat and prinomastat potently neutralised the procoagulant effects of *D. typus* venom (Fig. 1B). The IC_50_ values for marimastat and prinomastat were determined to be 34.2 nM (95% CI 24.2–48.5 nM) and 75.6 nM (95% CI 58.6–97.7 nM) respectively (Fig. 1C), demonstrating that marimastat was significantly more potent in this assay than prinomastat (p = 0.01). The PLA_2_ inhibitor varespladib (control non-SVMP inhibiting drug used throughout) had no neutralising effect on venom-induced coagulation at any of the tested drug concentrations (Supplemental File S1), a result in line with our expectations of procoagulant venom activity being mediated by SVMP toxins.

### 3.2 Inhibitory effects of small molecule drugs on nanofractionated *D. typus* venom coagulotoxins

**Fig. 2:**
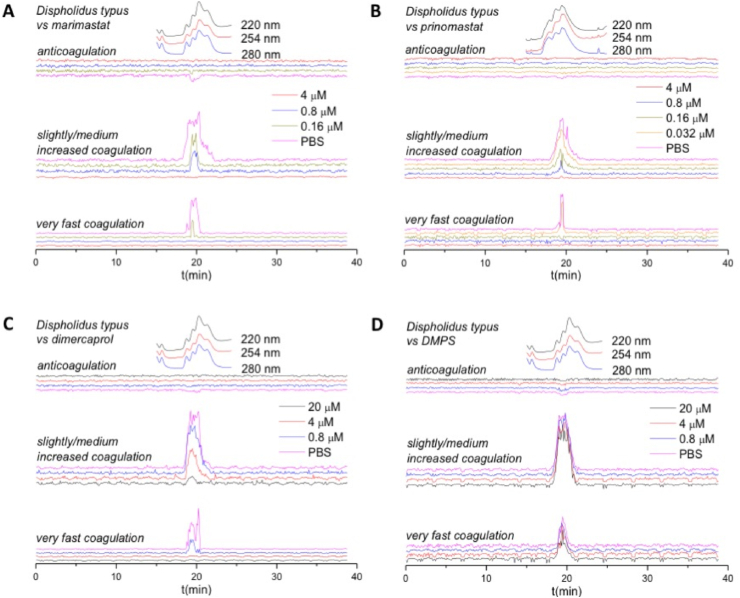
**Reconstructed coagulation chromatograms for nanofractionated *D. typus* venom toxins in the presence of different concentrations of A) marimastat, B) prinomastat, C) dimercaprol, and D) DMPS.** The negative peaks indicate anticoagulant activity where velocity is lower than the assay solution in control wells without venom toxins, and the positive peaks indicate procoagulant activity where velocity is higher than that in control wells without venom toxins. The top superimposed chromatograms are characteristic profiles of the UV trace at 220, 254 and 280 nm. PBS indicates venom only samples where PBS was used as a control for the inhibitors. Traces with different colours indicate different concentrations (final) of inhibitors in the assay.

To further characterise the coagulopathic activity of *D. typus* venom, and to better explore the specificity of the various small molecule inhibitors against specific toxins, we repeated the coagulation assay experiments using nanofractionated venom. As previously described, this method uses fractionated venom as the basis for measurements of the velocity of clotting in different wells in comparison to control wells, with procoagulant toxins producing positive peaks in the resulting bioassay chromatogram and anticoagulant toxins producing negative peaks (Still et al., 2017). In the venom-only analysis, broad positive peaks (18.4–22.0 min) were observed for both the ‘very fast coagulation’ chromatograms and the ‘slightly/medium increased coagulation’ chromatograms, indicative of an overall procoagulant effect of the venom. Detected bioactivities of *D. typus* venom were correlated with previously generated LC-MS and proteomics data (Slagboom et al., 2020). From this data, a candidate toxin mass of 23 kDa was identified for the procoagulant activity, which is within the range of SVMPs. The inhibitory effects of marimastat and prinomastat on nanofractionated *D. typus* venom toxins are depicted in Fig. 2A and B respectively. The peaks in the very fast and slight/medium increased coagulation chromatograms decreased with increasing concentrations of both marimastat and prinomastat, indicative of a dose-dependent restoration of normal clotting velocity. All coagulopathic activities were inhibited at 0.8 μM marimastat and 0.16 μM prinomastat for very fast coagulation chromatograms, and at 4 μM for both inhibitory molecules for slightly/medium increased coagulation activity. The inhibitory effects of dimercaprol and DMPS on nanofractionated *D. typus* venom toxins are depicted in Fig. 2C and D, respectively. By increasing the concentration range of dimercaprol, the procoagulant activity of *D. typus* venom was inhibited, with very fast coagulation activity fully inhibited at 4 μM, and slightly/medium increased coagulation activity at 20 μM. However, no substantial inhibition of procoagulant venom activity was observed with DMPS at any tested concentration up to 20 μM. These results reflect the considerable potency differences observed between the peptidomimetic inhibitors and the metal chelators in the crude venom plasma bioassays.

A very weak signal (peak centre at 19.6 min) was also detected in terms of anticoagulant venom activity. A previous study observed a much clearer negative anticoagulant peak with *D. typus* venom, though the venom was applied at a five-fold higher concentration (5.0 mg/mL venom) than that used in this study (1.0 mg/mL venom) (Slagboom et al., 2020). While varespladib has previously been demonstrated to be a potent inhibitor of anticoagulant venom activities induced by PLA_2_ toxins (Bittenbinder et al., 2018; Kazandjian et al., 2021; Youngman et al., 2020; Liu et al., 2021), in this study it produced no inhibitory effects on venom-induced coagulation, whether procoagulant or anticoagulant, at the maximal drug dose tested (20 μM) (Supplemental File S2). However, due to the weak anticoagulant venom activity observed in these experiments, the assay window for measuring such inhibition is limited.

### 3.3 Small molecule drugs inhibit crude *D. typus* venom SVMP activity

**Fig. 3:**
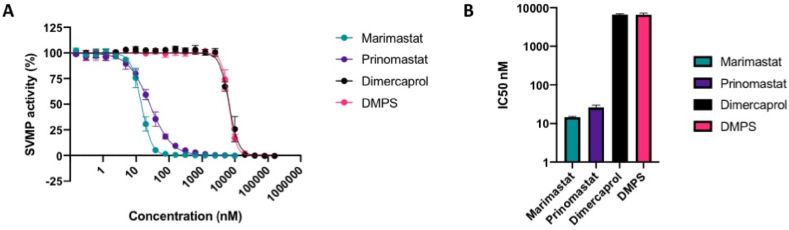
***In vitro* inhibition of *D. typus* crude venom SVMP activity by small molecule inhibitors. A)** SVMP activity of *D. typus* crude venom in the presence of marimastat (teal circles), prinomastat (dark purple circles), dimercaprol (black circles) and DMPS (pink circles) over a two-fold serial dilution curve, from which IC_50_ values were calculated. Data points represent the percentage of crude venom SVMP activity generated from the mean of four individual values recorded over two independent technical replicates, and error bars represent standard deviation. **B)** IC_50_ values of SVMP inhibition for marimastat, prinomastat, dimercaprol and DMPS.

To determine the specific inhibitory effects of the selected small molecule drugs on SVMP toxin activity, we performed IC_50_ screens of the five different toxin inhibitors in a previously defined kinetic enzymatic SVMP assay using crude *D. typus* venom. As previously observed (Alomran et al., 2021), *D. typus* venom demonstrated strong SVMP-specific activity in this assay. The inhibitory effects of the matrix metalloproteinase inhibitors marimastat and prinomastat and the metal chelators DMPS and dimercaprol against the venom SVMP activity of *D. typus* are displayed in Fig. 3. Marimastat and prinomastat demonstrated nanomolar IC_50_ values of 14.5 (95% CI 13.9–15.2 nM) and 25.9 nM (95% CI 22.6–29.7 nM) respectively, and complete inhibition of venom activity at 156 nM for marimastat and 2.5 μM for prinomastat (Fig. 3A). Inhibition of SVMP activity by dimercaprol and DMPS as measured by IC_50_ values was significantly lower than that observed for marimastat and prinomastat (p < 0.02 for all comparisons). Dimercaprol and DMPS demonstrated highly comparable inhibition of *D. typus* SVMP activity with complete inhibition obtained at 40 μM and IC_50_ values of 6.68 (95% CI 6.36–7.02 μM) and 6.61 μM (95% CI 6.38–6.87 μM), respectively (Fig. 3B), with no significant differences between the two IC_50_ values. As anticipated, the control drug used in this study, the PLA_2_ inhibitor varespladib, showed no inhibitory activity at any of the concentrations tested (maximum concentration 10 μM) (Supplemental File S3).

### 3.4 Marimastat and DMPS provide some protection against *D. typus* venom-induced lethality *in vivo*

**Fig. 4:**
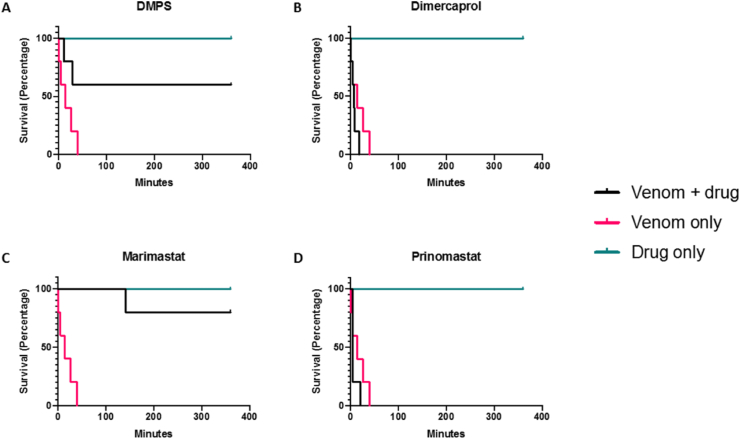
**The small molecule drugs marimastat and DMPS significantly increase the survival times of mice receiving lethal doses of *D. typus* venom.** Data shown as Kaplan-Meier survival graphs for experimental animals (n = 5 per group, except prinomastat only group where n = 4) treated with either: 90 μg (4 x LD_50_) of *D. typus* venom only (magenta), 118 μg of drug only (cyan) or 90 μg venom and 118 μg drug (black). Treatments were pre-incubated at 37 °C for 30 minutes prior to intravenous injection via the tail vein and animals were monitored for 6 hours. Data is shown for: **A)** DMPS, **B)** dimercaprol, **C)** marimastat, and **D)** prinomastat.

Given that inhibition of SVMP and coagulotoxic activities *in vitro* have previously been demonstrated to translate into varying degrees of *in vivo* protection against systemic envenoming (Albulescu et al., 2020a, 2020b) we next tested the capability of the four SVMP-inhibiting small molecule drugs to protect against *D. typus* venom-induced lethality *in vivo*. To do so, we used a modified version of the WHO-recommended protocol of murine venom neutralisation (ED_50_ assay). All five experimental animals treated intravenously with 4 x LD_50_ doses of *D. typus* venom (90 μg) succumbed to the lethal venom effects within the first hour of the experiment (mean 17 minutes, range 1–40 minutes), as shown in Fig. 4. The intravenous co-delivery of the small molecule drugs preincubated with *D. typus* venom revealed that both prinomastat and dimercaprol failed to protect against venom-induced lethality at the single therapeutic dose tested (118 μg), with all experimental animals in these groups succumbing to venom lethality within the first 30 minutes (mean 7.8 minutes for both groups; prinomastat range 4–21 minutes; dimercaprol range 1–18 minutes), in a highly comparable manner to the venom only control. Contrastingly, DMPS and marimastat both showed a significant degree of protection against *D. typus* venom-induced lethality. Three of the five experimental animals dosed with DMPS were protected for the duration of the experiment (6 hours), with two deaths occurring within the first hour, resulting in a mean survival time of 224 minutes compared to 17.2 minutes in the venom only control group (log-rank test, p = 0.047). Of the animals dosed with marimastat, four of the five animals were protected for the duration of the experiment, with mean survival times of 316.2 minutes compared to 17.2 minutes in the venom only control group (log-rank test, p = 0.002). The single non-surviving experimental animal in this group was euthanised at 141 minutes. No adverse effects were observed in experimental drug -only control animals and, consequently, all survived the duration of the experiment (Fig. 4).

## 4 Discussion

Conventional animal-derived antivenom, although a life-saving treatment, has numerous deficiencies that impair its utility in the treatment of snakebite. Although a seemingly effective antivenom for treating *D. typus* envenoming is available in South Africa, it is often difficult to source in other regions of the continent and can be catastrophically unaffordable for patients (Krüger and Lemke, 2019), and this scenario encapsulates the challenges faced by snakebite victims the world over. There is therefore an urgent need to develop alternative/supplementary therapeutics that are stable, effective, affordable and available in remote rural areas where medical access is limited. Small molecule inhibitors that can broadly neutralise a class of key toxins in snake venom following oral administration are possible solutions in this regard (Clare et al., 2021) and Phase II clinical trials for small molecule inhibitors of snakebite are underway (ClinicalTrials.gov, 2021). Rapid-onset pathologies such as VICC, together with tissue damage induced by SVMPs, are only partially neutralised by antibody based antivenoms (Gutiérrez et al., 2006), which suffer from poor tissue distribution due to the inherent large size of antibodies (Gutiérrez et al., 2017). By contrast, the drastically smaller size of the inhibitors tested in this study enables favourable properties of rapid and effective tissue penetration and potential for oral delivery, due to their pharmacokinetic and physicochemical properties (Lewin et al., 2018a, 2018b; Kini et al., 2018). Moreover, repurposing these small molecule inhibitors that are either licensed drugs or phase I-approved drug candidates could significantly shorten drug development times as safety profiles, pharmacokinetics, bioavailability and tolerance data on these molecules have already been attained (Kini et al., 2018; Lewin et al., 2016; Knudsen et al., 2019). Current evidence of the utility of small molecule inhibitors against snakebite indicates that they may be particularly effective as first line, early intervention therapeutics and/or bridging therapies for initial and adjunct treatment in community settings, before patients are able to access antivenom in healthcare centres (Bulfone et al., 2018; Howes et al., 2007; Lewin et al., 2016).

In this study we assessed the ability of four small molecule inhibitors to neutralise *D. typus* venom toxin activities *in vitro* and *in vivo.* In line with previous findings (Debono et al., 2017, 2020; Ainsworth et al., 2018; Pla et al., 2017), our study demonstrates that *D. typus* venom toxicity is largely conferred by SVMP toxins, which are likely responsible for causing coagulopathy *in vivo*. Our data from *in vitro* assays of *D. typus* venom activity demonstrate that the matrix metalloproteinase inhibitors marimastat and prinomastat are potent inhibitors of the SVMP-mediated procoagulant effects of this venom, with both compounds demonstrating similar inhibitory activity in *in vitro* assays of plasma coagulation and SVMP activity. Indeed, marimastat and prinomastat showed nanomolar IC_50_ values in the crude venom plasma coagulation assay and crude venom SVMP assay, and both drugs showed inhibitory effects at low micromolar concentrations in the plasma coagulation assay with nanofractionated venom. However, and in contrast with *in vitro* SVMP-inhibiting prowess, in the *in vivo* murine models of envenoming assays, all animals in the prinomastat group succumbed to lethality from *D. typus* venom, whilst an equivalent amount of marimastat conferred 80% protection. This was unexpected and hints at different levels of drug exposure and metabolism in this single dose intravenous-delivered model, as both matrix metalloproteinase inhibiting drugs showed potent inhibitory activity in our *in vitro* assays and have been shown in other studies to neutralise the *in vivo* lethal effects of *E. ocellatus* venom in murine models (Arias et al., 2017; Albulescu et al., 2020a). Studies by other groups have similarly shown the impressive *in vitro* inhibitory activities of marimastat and prinomastat against SVMP activity in rattlesnakes and a wide range of palearctic vipers (Chowdhury et al., 2021a; Seneci et al., 2021) and strong inhibitory effects of prinomastat against the anticoagulant activity of spitting cobra venoms (Chowdhury et al., 2021b).

The metal chelators dimercaprol and DMPS demonstrated lower potency than marimastat and prinomastat in the *in vitro* studies of SVMP activity and plasma coagulation, which is in agreement with previous studies (Albulescu et al., 2020a, 2020b; Chowdhury et al., 2021a), whilst the PLA_2_ inhibitor varespladib, used as a non-SVMP inhibiting control, produced no inhibitory effects on the venom. Of the two metal chelators, DMPS showed the weakest inhibitory activity in *in vitro* assays of venom bioactivity. Despite this reduced *in vitro* potency in comparison with the peptidomimetic matrix metalloproteinase inhibitors, DMPS conferred a degree of protection against venom lethality in murine models of envenoming, with significant increases in mean survival times and 60% of mice surviving until the end of the experimental time window. None of the experimental animals dosed with the other metal chelator, dimercaprol, survived the experiment, despite the comparable mechanism of action and *in vitro* inhibitory potency in the coagulation and SVMP assays. These results contrast with our previous preclinical study investigating *E. ocellatus* envenoming, which found that both dimercaprol and DMPS provided protection against lethal effects in this same intravenous murine model, though are consistent with DMPS exhibiting superior preclinical efficacy (Albulescu et al., 2020a).

The notable discrepancies between the *in vitro* and *in vivo* experiments described herein exemplifies the complexity associated with relying on *in* *vitro* potency-based screens as a means to predict the efficacy of small molecule drugs in *in vivo* experiments. While efficacy data gained from *in vitro* experiments is undoubtedly an essential prerequisite prior to preclinical efficacy testing, substantial differences in inhibitor potency at this step does not preclude preclinical efficacy, which ultimately is dictated by drug exposure. Equally, the preclinical model utilised here, consisting of the pre-incubation of drug with venom followed by intravenous co-delivery, is largely detached from the clinical scenario of a snakebite. While this is the WHO-recommended method for preclinical assessment of antivenom efficacy, and thus is a logical starting point for assessing preclinical efficacy, this method does not reflect the biodistribution of venom during early envenoming and uses a non-clinically relevant route of venom injection. The pharmacokinetics of snake envenoming demonstrate that venoms undergo an initial absorption phase when administered through the intramuscular or subcutaneous routes, which are more representative of a snakebite, whereas the intravenous route bypasses the absorption phase to the distribution phase and finally the elimination phase (Sanhajariya et al., 2018). Furthermore, the intravenous pre-incubation assay does not take into account the pharmacokinetics/pharmacodynamics of the unbound test inhibitor (Gutiérrez et al., 2021). Thus, the lack of efficacy observed with prinomastat (compared with marimastat) here, for example, may be the result of a lack of *in vivo* dose optimisation and thus sub-optimal exposure. Further work is required to better define the pharmacokinetic and pharmacodynamic profiles of small molecule drugs in preclinical models of snakebite envenoming to inform the design of optimised preclinical dosing regimens applicable for use in more biologically-realistic models of envenoming (e.g. “challenge then treat models”) (Albulescu et al., 2020a, 2020b; Lewin et al., 2018a; Gutiérrez et al., 2017; Knudsen et al., 2020), and taking into account plasma stability, drug metabolism, and appropriate compartmental models of venom and small molecule inhibitors.

In sub-Saharan Africa, VICC is only known to be commonly caused by *Echis* spp. and *D. typus,* and the venoms of these snakes have been shown to converge on similar SVMP-rich venom composition profiles (Ainsworth et al., 2018), suggesting that *D. typus* venom may be amenable to neutralisation by previously identified inhibitors of *Echis* venoms. This study investigated the ability of repurposed small molecule inhibitors to effectively neutralise *D. typus* venom activity *in vitro* and *in vivo,* and identified the SVMP inhibiting drugs DMPS and marimastat as two lead compounds that provide a significant degree of preclinical protection against the lethal effects of *D. typus* venom. Previous studies have demonstrated that both DMPS and marimastat also provide preclinical action against *E. ocellatus* venom (Albulescu et al., 2020a, 2020b), and the present study may expand the range of snake species and, consequentially, the number of snakebite victims of that could potentially benefit from receiving an early intervention with small molecule therapeutics. While considerable work remains to be done before such toxin inhibitors can progress into snakebite clinical trials, our findings here provide a strong rationale to continue such work, and ultimately progress towards the long term future goal of clinically evaluating the efficacy of such small molecule drugs in all cases of diagnostically indicated VICC following snakebite envenoming in sub-Saharan Africa.

## Credit author statement

Stefanie K Menzies: Methodology, Investigation, Formal analysis, Data curation, Writing – original draft, Writing – review & editing, Visualization. Rachel H Clare: Methodology, Investigation, Formal analysis, Data curation, Writing – original draft, Writing – review & editing, Visualization, Funding acquisition. Chunfang Xie: Methodology, Investigation, Formal analysis, Data curation, Writing – original draft, Writing – review & editing, Visualization, Funding acquisition. Adam Westhorpe: Investigation, Formal analysis, Data curation, Writing – review & editing. Steven R Hall: Investigation, Writing – review & editing. Rebecca J Edge: Investigation, Writing – review & editing. Jaffer Alsolaiss: Investigation, Writing – review & editing. Edouard Crittenden: Investigation, Writing – review & editing. Amy E Marriott: Investigation, Writing – review & editing. Robert A Harrison: Investigation, Writing – review & editing. Jeroen Kool: Conceptualization, Writing – review & editing, Supervision, Funding acquisition. Nicholas R Casewell: Conceptualization, Investigation, Writing – original draft, Writing – review & editing, Supervision, Funding acquisition.

## Funding

This research was funded in whole, or in part, by the 10.13039/100010269Wellcome Trust, grant numbers as detailed below. For the purpose of open access, the authors have applied a CC BY public copyright licence to any Author Accepted Manuscript version arising from this submission. R.H.C. acknowledges funding support from the Director's Catalyst Fund at 10.13039/100014976LSTM [supported by Wellcome Institutional Strategic Support Fund 3 (204806/Z/16/Z) and LSTM Internal Funding]. N.R.C. acknowledges a UK Medical Research Council research grant (MR/S00016X/1) and a Sir Henry Dale Fellowship (200517/Z/16/Z) jointly funded by Wellcome and the Royal Society. N.R.C and J.K. acknowledge funding provided by a Wellcome project grant (221712/Z/20/Z). C.X. acknowledges funding support from the China Scholarship Council (CSC) fellowship (201706250035).

## Ethics statement

All animal experiments were performed using protocols approved by the Animal Welfare and Ethical Review Boards of the Liverpool School of Tropical Medicine and the University of Liverpool, under project licence (P58464F90) approved by the UK Home Office in accordance with the UK Animal (Scientific Procedures) Act 1986.

## Declaration of competing interest

The authors declare the following financial interests/personal relationships which may be considered as potential competing interests: Rachel H Clare reports financial support was provided by Wellcome Trust. Jeroen Kool reports financial support was provided by 10.13039/100010269Wellcome Trust. Nicholas R Casewell reports financial support was provided by 10.13039/100010269Wellcome Trust. Nicholas R Casewell reports financial support was provided by Medical Research Council. Nicholas R Casewell reports financial support was provided by The Royal Society. Chunfang Xie reports financial support was provided by China Scholarship Council.
